# Validation of the Social Media Disorder Scale in Adolescents: Findings From a Large-Scale Nationally Representative Sample

**DOI:** 10.1177/10731911211027232

**Published:** 2021-06-30

**Authors:** Maartje Boer, Gonneke W. J. M. Stevens, Catrin Finkenauer, Ina M. Koning, Regina J. J. M. van den Eijnden

**Affiliations:** 1 Utrecht University, Utrecht, Netherlands

**Keywords:** problematic social media use, social media addiction, adolescents, psychometric properties, validation study

## Abstract

Large-scale validation research on instruments measuring problematic social media use (SMU) is scarce. Using a nationally representative sample of 6,626 Dutch adolescents aged 12 to 16 years, the present study examined the psychometric properties of the nine-item Social Media Disorder scale. The structural validity was solid, because one underlying factor was identified, with adequate factor loadings. The internal consistency was good, but the test information was most reliable at moderate to high scores on the scale’s continuum. The factor structure was measurement invariant across different subpopulations. Three subgroups were identified, distinguished by low, medium, and high probabilities of endorsing the criteria. Higher levels of problematic SMU were associated with higher probabilities of mental, school, and sleep problems, confirming adequate criterion validity. Girls, lower educated adolescents, 15-year-olds, and non-Western adolescents were most likely to report problematic SMU. Given its good psychometric properties, the scale is suitable for research on problematic SMU among adolescents.

Social network sites and instant messengers such as Instagram and Snapchat have become prominent parts of adolescents’ lives (Anderson & Jiang, 2018). The social involvement and entertainment that are associated with social media use (SMU) may enhance adolescents’ social capital and feelings of connectedness (Verduyn et al., 2017). However, SMU can become concerning when it is associated with addiction-like symptoms, such as a loss of control over SMU (Griffiths et al., 2014), which we refer to as *problematic SMU*. Research has shown that adolescent problematic social media users are more likely to experience mental health problems (Marino et al., 2018; Van den Eijnden et al., 2018), have lower school achievements (Al-Menayes, 2015; Vangeel et al., 2016), and lower sleep quality (Andreassen et al., 2012, Wong et al., 2020). While these studies emphasize the potential threat of problematic SMU to adolescents’ development and daily life functioning, validation work on instruments that measure problematic SMU is limited. The present study aims to validate the nine-item Social Media Disorder (SMD) scale (Van den Eijnden et al., 2016) in a Dutch nationally representative adolescent sample.

There has been debate for many years about whether heavy engagement in activities, for example in SMU, should be regarded as addictive behaviors (Kardefelt-Winther et al., 2017; Van Rooij et al., 2018). For a long time, diagnostic manuals have linked “addiction” to substance-related disorders only (Potenza, 2014). However, it has been put forward that all addictive behaviors, either related to substances or behaviors, result from similar individual biological and psychosocial processes and share six core criteria of addiction (Griffiths, 2005; Potenza, 2014). These core criteria are: *salience* (i.e., preoccupation: constantly thinking about the activity in concern), *mood modification* (i.e., escape: the activity helps find relief from negative feelings), *tolerance* (i.e., wanting to engage in the activity more and more), *withdrawal* (i.e., experiencing unpleasant physical or emotional effects when the activity is not possible), *conflict* (i.e., having conflicts at school, work, or with personal close relationships due to the heavy engagement in the activity), and *relapse* (i.e., persistence: being unable to stop or to control the activity; Griffiths, 2005). With the increasing evidence demonstrating the similarities between substance-related disorders and gambling and gaming disorders, the latest version of the *Diagnostic and Statistical Manual of Mental Disorders* (*DSM-5*) added gambling disorder to the “substance-related and addictive disorders” category and internet gaming disorder as a condition requiring further study, whereby both behavioral addictions are defined by the core criteria of addiction and a few additional criteria (American Psychiatric Association [APA], 2013). Unlike gambling and gaming disorder, the *DSM-5* does not acknowledge SMD as a (tentative) behavioral addiction. However, SMU is a relatively new behavior, which increased especially after the rise of smartphone use around 2012 (Twenge et al., 2018), when the development of the *DSM-5* was already in progress. It generally takes several decennia before disorder classification systems acknowledge the existence of new disorders. Scholars argue that people can experience SMU-related addiction symptoms that parallel substance-related addiction symptoms, and that social media addiction results from the same “biopsychosocial” processes that drive substance-related addictions (Griffiths, 2013; Griffiths et al., 2014; Kuss & Griffiths, 2011). Furthermore, there is increasing evidence that the presence of these symptoms impair adolescents’ cognitive and psychosocial functioning (Boer et al., 2020; Boer et al., 2021; Van den Eijnden et al., 2018). In absence of a formal recognition of SMD as a behavioral addiction, we refer to it as “problematic SMU.”

Researchers have used several instruments to measure problematic SMU, but most instruments have not been validated (Andreassen, 2015). To our knowledge, the only instrument that has been validated in a large-scale representative adolescent sample is the Bergen Social Media Addiction Scale (BSMAS; Andreassen et al., 2016; Bányai et al., 2017; Lin et al., 2017). The BSMAS has been developed parallel to the SMD scale, and covers the six core criteria of addiction (Griffiths, 2005; Griffiths et al., 2014). Scholars have argued that the presence of addiction criteria in relation to (social media) behaviors is not necessarily indicative of whether the behavior is harmful, which is considered a crucial aspect for defining addiction-like behaviors (Kardefelt-Winther et al., 2017; Van Rooij et al., 2018). Therefore, the SMD scale measures the same six core criteria of addiction and two additional criteria that refer to harmful implications due to SMU: *problems* (i.e., experiencing problems on important life domains due to SMU) and *displacement* (i.e., displacing social or recreational activities by SMU). The SMD scale also includes *deception* (i.e., lying about time spent on SMU). These nine criteria for problematic SMU were adopted from the *DSM-5* definition of internet gaming disorder (APA, 2013; Lemmens et al., 2015). By adding three additional criteria to the six core criteria of addiction, the nine-item SMD scale provides a more comprehensive operationalization of problematic SMU.

The SMD scale was developed based on a confirmatory factor analysis (CFA) on data from a 27-item questionnaire assessed among 10- to 17-year-old Dutch adolescents, which included three items for each of the nine criteria (Lemmens et al., 2015; Van den Eijnden et al., 2016). The nine-item SMD scale consists of the items that showed the highest factor loading per criterion. The nine items can be regarded as nine subdimensions, yet together, they intend to reflect one overarching dimension (Van den Eijnden et al., 2016). Indeed, CFA on the nine-item scale demonstrated solid structural validity for a unidimensional (i.e., one-factor) model, with acceptable internal consistency of the test scores. Also, higher scores were associated with higher reports of compulsive internet use, self-declared social media addiction, and mental health problems, indicating good convergent and criterion validity of the test score interpretations (Van den Eijnden et al., 2016). An adapted version of the SMD scale with polytomous instead of dichotomous response scales was validated among a sample of 553 Turkish adolescents aged 14 to 18 years (Savci et al., 2018). In this study, exploratory factor analysis (EFA) also identified one dimension, and internal consistency of the test scores was acceptable. Also, the convergent and criterion validity of the test score interpretations was adequate (Savci et al., 2018). Although these studies indicated that the SMD scale has appropriate psychometric properties, important validation steps remain unaddressed.

First, the structural validity of the SMD scale score interpretations has not been explored in a nationally representative sample. Although the scale aims to measure one overarching dimension problematic SMU (Van den Eijnden et al., 2016), exploring possible multidimensionality is crucial to enhance our understanding of problematic SMU. Furthermore, the use of the sum-score of the nine items to assess adolescents’ level of problematic SMU is only justified when the scale measures one underlying dimension to which all nine items substantially contribute. Second, although the test scores of the SMD scale were found to have acceptable internal consistency (Savci et al., 2018; Van den Eijnden et al., 2016), the reliability at different levels of problematic SMU has not been investigated. Third, it remains unclear whether the factor structure of the SMD scale is equal across subpopulations, which is required to reliably compare observed levels of problematic SMU across subpopulations (Chen, 2007). Because studies suggest that girls, low-educated adolescents, specific age groups, and immigrant adolescents are more sensitive to developing problematic SMU (Bányai et al., 2017; Ho et al., 2017; Mérelle et al., 2017), it is pivotal to examine whether the scale is measurement invariant across these groups in order to be able to interpret these differences. Fourth, research shows that it is often possible to distinguish subgroups whose members show similar characteristics with regard to a particular behavior (Bányai et al., 2017; Király et al., 2017; Lemmens et al., 2015; Peeters et al., 2019). It has not been investigated whether the SMD scale can be used to study subgroups of users, and if so, by which set of criteria these subgroups could be characterized. The identification of such subgroups may provide more insight into the phenomenon of problematic SMU and allow researchers to use the scale to compare subgroups of users on, for example, their well-being. Fifth, previously conducted criterion validity analyses on the SMD scale were limited to assessments of mental health problems (Savci et al., 2018; Van den Eijnden et al., 2016). In order to verify whether the test score interpretations of the scale are valid, associations with other constructs related to adolescents’ daily life functioning should be considered as well, including school functioning and sleep problems.

## Current Study

Given the increasing body of literature showing that problematic SMU is negatively associated with mental health and functioning in important life domains, it is essential that research on problematic SMU uses a psychometrically sound instrument. The present study is the first that uses a large-scale, nationally representative sample of adolescents to validate the nine-item SMD scale. Data came from 6,626 Dutch secondary school adolescents aged 12 to 16 years who participated in the Health Behavior in School-Aged Children study (HBSC). The present study aimed to investigate the (1) structural validity, (2) reliability, (3) measurement invariance, (4) item score patterns, and (5) criterion validity of the SMD scale scores. After these validation steps, we examined the association between adolescents’ demographic characteristics and problematic SMU.

## Method

### Sample

Analyses were carried out using cross-sectional data from the HBSC study conducted in the Netherlands. The study is part of a WHO-collaborative cross-national study carried out every 4 years since 1983 and investigates adolescents’ well-being and health behaviors in their social context. We used the Dutch HBSC sample collected in 2017 among secondary school students (*n* = 6,718; Stevens et al., 2018). The sample consisted of adolescents (51.16% boys) aged 12 to 16 years (*M*_age_ = 13.94, *SD*_age_ = 1.37). The sample comprised different educational levels (46.32% pre-vocational, 25.34% general higher, and 28.34% pre-university) and ethnic backgrounds (78.27% native, 16.59% had at least one parent born in a non-Western country, and 5.15% had at least one parent born in a non-Dutch Western country). Although the sample closely resembled the adolescent population in the Netherlands, the data included sample weights to adjust for sample distribution differences with the population. These weights included gender, educational level, school year, and urbanization degree of participants. The HBSC-sample was therefore nationally representative for the Dutch adolescent population in secondary schools (Van Dorsselaer, 2018). For analytic purposes, the sample was randomly split into two subsamples, which we labelled as “calibration sample” (*n =* 3,359) and “validation sample” (*n =* 3,359). Respondents who did not respond to any of the items on the SMD scale were excluded from these samples (*n* = 92), which yielded a final sample of 6,626 (*n*_calibration_ = 3,310, *n*_validation_ = 3,316).

The HBSC-data had a hierarchical structure, where adolescents were nested in school classes (*n* = 328) and schools (*n* = 85). Schools were randomly selected from a list of schools provided by the Dutch Ministry of Education, after which three to five classes per school (depending on the number of students per school) were randomly selected. The response rate on school-level was 37%. The main reason for not participating was that schools were already approached for other research. School nonresponse was somewhat higher among schools in urban than in rural areas, χ^2^(5) = 15.6, *p* < .01. Participating and nonparticipating schools did not differ regarding their average number of students and ethnic composition. There were no refusals on school class level, and on the individual level, 92% of all selected adolescents participated. The individual nonresponse was mostly related to absence from school at the day of survey assessment, due to, for example, illness or truancy (Van Dorsselaer, 2018).

Participation in the HBSC-study was voluntary and anonymous, conducted through digital self-completion questionnaires during school hours monitored by trained research assistants. School principals sent information about the study to all parents of adolescents in the selected school classes in advance, and parents were provided the opportunity to refuse participation. Almost all parents provided this passive consent (>99%). Adolescents gave active consent by ticking a box at the start of the survey that confirmed their approval (>99%). The study was approved by the ethics council of Social Sciences of Utrecht University (FETC17-079).

### Measures

#### Problematic SMU

The SMD scale was used to measure problematic SMU (Van den Eijnden et al., 2016). The scale consists of nine dichotomous items corresponding to the nine diagnostic criteria for internet gaming disorder as stated in the appendix of the *DSM-5* (APA, 2013; Lemmens et al., 2015). The questionnaire was introduced with: “We are interested in your experiences with social media. The term social media refers to social network sites (e.g., Facebook, Twitter, Instagram, Google+, Pinterest) and instant messengers (e.g., WhatsApp, Snapchat, Facebook messenger).” Subsequently, adolescents were asked, “During the past year, have you ( . . . ),” followed by, for example, “regularly found that you can't think of anything else but the moment that you will be able to use social media again?” (preoccupation). Response options were (1) *yes* and (0) *no*. The items “displacement” and “escape” had slightly different wordings than the initial scale (Van den Eijnden et al., 2016).

#### Mental Health Problems

Four subscales of the self-report Strength and Difficulties Questionnaire were used to measure mental health problems, including *emotional problems, conduct problems, hyperactivity*, and *peer problems* (Goodman et al., 2003). Each subscale consists of five items, for example, “I worry a lot” (emotional problems), “I am often accused of lying and cheating” (conduct problems), “I am easily distracted, I find it difficult to concentrate” (hyperactivity), and “Other children or young people pick on me or bully me” (peer problems). Answer categories were (0) *not true*, (1) *somewhat true*, and (2) *certainly true.* Given the ordinal nature of the items, internal consistency of the test scores of each subscale was calculated using the ordinal alpha based on the polychoric correlation matrix (Gadermann et al., 2012). Ordinal alpha was .81 for emotional problems, .67 for conduct problems, .76 for hyperactivity, and .64 for peer problems. Our aim was to study the associations between problematic SMU and problematic levels of mental health problems. Therefore, subscale sum scores were dichotomized in line with recommendations from Goodman et al. (2003): Subscale sum scores higher than the 80th percentile were coded as (1) *borderline or abnormal*, whereas subscale sum scores lower than the 80th percentile were coded as (0) *normal*.

#### School Problems

Adolescents were asked how they feel about school at present, with response ranging from (1) *I like it a lot* to (4) *I don’t like it at all* (Inchley et al., 2016). In order to study associations with particularly school dissatisfaction, the variable was recoded into a dichotomous variable *school dissatisfaction*, with categories (1) *I don’t like it very much/I don’t like it at all* and (0) *I like it a lot*/*I like it a bit*. Adolescents were also asked whether they feel pressured by the schoolwork they have to do, with responses ranging from (1) *not at all* to (4) *a lot* (Inchley et al., 2016). To study the association between problematic SMU and schoolwork pressure, this variable was dichotomized into the variable *perceived school pressure*, with categories (1) *some/a lot* and (0) *not at all/a little*.

#### Sleep Problems

Adolescents were asked what time they usually go to bed and what time they usually wake up on schooldays. Answers on these questions were used to establish whether the reported average sleep duration met the age-specific recommendation for daily sleep duration according to the National Sleep Foundation (Hirshkowitz et al., 2015). For 12- and 13-year-olds, at least 9 hours of sleep is recommended, whereas for 14- until 16-year-olds, at least 8 hours of sleep is recommended. In order to study the association between problematic SMU and low sleep duration specifically, we created a dichotomous variable *lower sleep duration than recommended*, with categories (1) *not meeting the recommendation* and (0) *meeting the recommendation*. Also sleep quality was measured using five items from the Groningen Sleep Quality Scale (Meijman et al., 2006). Adolescents were asked to evaluate their sleep during the past week on schooldays, for example “I felt like I slept poorly last night.” Responses ranged from (1) *never* to (5) *(almost) always*, and therefore high values indicated lower sleep quality. The test scores of the five items yielded a Cronbach’s alpha from .77. The mean of the five items was dichotomized into the variable *low sleep quality* with categories (1) *mean score above 3.5* and (0) *mean score below 3.5*.

#### Demographic Characteristics

*Gender* consisted of two categories: (1) *girl* and (0) *boy*. The Dutch education system distinguishes broadly three paths of secondary education: pre-vocational education (“VMBO”), general secondary education (“HAVO”), or pre-university education (“VWO”). Students typically follow one of the three paths. Hence, *educational level* consisted of categories (1) *low* (pre-vocational education, i.e., all “VMBO” levels or “VMBO/HAVO”), (2) *medium* (general higher education, i.e., “HAVO” or “HAVO/VWO”), and (3) *high* (pre-university education, i.e., “VWO”). *Age* varied from 12- to 16-year-old. *Ethnic background* was determined by adolescents’ responses to the question where their parents were born, and consisted of three categories: *native* (both parents born in the Netherlands), *non-Western* (at least one parent from Africa, Latin-America, Asia [excluding Indonesia and Japan] or Turkey), and *other Western* (at least one parent from Europe [excluding Turkey], North-America, Oceania, Indonesia, or Japan, and no parent from a non-Western country; CBS, 2019).

### Analysis Strategies

#### Structural Validity

We explored the number of underlying factors measured by the SMD scale by conducting an EFA using the calibration sample. A factor should consist of at least three items to be considered as a reliable factor (Costello & Osborne, 2005; Fabrigar et al., 1999). Therefore, with nine items on the scale, we decided a priori that a maximum of three factors should be extracted in the EFA. An oblique (goemin) rotation was applied to interpret the factor loadings, which assumed that factors in the multiple factor solution were correlated. The EFA-factor solutions were evaluated based on the empirical eigenvalues, Horn’s parallel analysis, model fit, and quality. The number of factors with *empirical eigenvalues* higher than one indicated the number of factors to extract. *Parallel analysis* evaluated this solution by comparing the empirical eigenvalues with 1,000 randomly generated eigenvalues based on the same number of variables and sample size. The number of factors to retain was indicated by the number of factors where the 95th percentile random data eigenvalues did not exceed the empirical eigenvalues (Ledesma & Valero-Mora, 2007). *Model fit* of the factor solution was assessed using the comparative fit index (CFI), Tucker–Lewis index (TLI), root mean square error of approximation (RMSEA), and standardized root mean square residual (SRMR; Schermelleh-Engel et al., 2003). We did not rely on the χ^2^ statistic given its sensitivity to large sample sizes (Fabrigar et al., 1999). *Quality* of the factor solutions was considered poor when removal of items with factor loadings below .5 or with cross-loadings that differed by less than .2 yielded factors with less than three items (Costello & Osborne, 2005; Howard, 2016). To examine the robustness of the EFA results, we conducted Velicer’s minimum average partial (MAP) analysis using the calibration sample. This analysis evaluates multiple factor solutions based on principal component analysis by calculating the average partial correlation between items when the first component is partialled out, when the first two components are partialled out, and so on. The number of factors to retain was indicated by the number of components where the average partial correlation was at its minimum (Velicer, 1976).

To examine the robustness and generalizability of the findings from the EFA and MAP analyses, the obtained factor solution was evaluated with a CFA using the validation sample.

#### Reliability and Item Performance

Given the dichotomous nature of the nine items, reliability of the scores was calculated using the ordinal alpha based on the tetrachoric correlation matrix (Gadermann et al., 2012), which indicates the level of internal consistency. Reliability was further analyzed using item response theory (IRT). IRT models describe the relation between observed item scores and their underlying unobserved latent trait (θ) by means of difficulty (i.e., threshold) and discrimination (i.e., loading) parameters (Baker, 2001). The difficulty parameter of an item indicates at which value of θ respondents have a 50% probability of endorsing that item. The discrimination parameter of an item denotes the item’s ability to discriminate between respondents with high versus low values on the continuum of θ, with higher values suggesting better discrimination (Baker, 2001). The difficulty and discrimination parameters were used to generate information curves, which graphically illustrate the amount of information that was provided by single items and the total scale across the continuum of θ. The higher the information, the higher the reliability (Toland, 2014).

#### Measurement Invariance

Multigroup CFAs were conducted to examine whether the factor structure of the SMD scale was measurement invariant across gender, educational level, age, and ethnic background. First, *configural invariance* was modelled by fitting a multigroup CFA where all item loadings and thresholds were freely estimated across groups (e.g., across boys and girls). Second, *scalar invariance* was modelled by fitting a multigroup CFA where item loadings as well as item thresholds were constrained to be equal across groups. The models were estimated according to specific guidelines for invariance testing of dichotomous variables, which do not allow for a separate test of *metric invariance* (i.e., multigroup CFA with equal factor loadings and free thresholds) due to model nonidentification (Muthén & Muthén, 2017b). Measurement invariance was established when adding the equality constraints did not substantially deteriorate model fit in terms of CFI, RMSEA, and SRMR (Chen, 2007). These fit indices are commonly used in measurement invariance analyses on large samples as an alternative to χ^2^-difference tests (Chen, 2007).

#### Subgroups of Users

We explored whether we could identify subgroups with specific item score patterns by means of Latent Class Analysis (LCA) on the nine items. Specifically, we evaluated different class (i.e., subgroup) solutions on their model fit and classification accuracy (Nylund et al., 2007). *Model fit* was examined using the Akaike information criterion (AIC), Bayesian information criterion (BIC), and the Lo-Mendell-Rubin adjusted likelihood ratio test (LMR-LRT). *Classification accuracy* was based on the entropy. After the best class solution was established, we compared adolescents’ observed item scores across the empirically identified classes. In addition, the LCA-models assume by default that the items are independent within each class, that is, that there are no correlations between the residuals of the items (Asparouhov & Muthén, 2015). This assumption of “conditional independence” is often too restrictive, because it typically does not comply with the data. Therefore, imposing the assumption may lead to biased results and wrong model selection (Uebersax, 1999). Hence, a sensitivity analysis was conducted where the LCA was repeated while allowing for conditional dependence. Particularly, for each model, we consulted the “bivariate fit information” to inspect the pairs of items that violated the assumption based on the bivariate Pearson chi-square (>10), after which we modified the respective model by adding correlations between the pairs of items that violated the assumption (Asparouhov & Muthén, 2015). We applied this procedure to all class solutions and evaluated whether it yielded a similar model selection as the initial analysis that assumed conditional independence.

#### Criterion Validity

Criterion validity defines the extent to which test scores relate to outcomes they should theoretically be related to. We examined whether higher levels of problematic SMU were associated with more mental health problems (emotional problems, conduct problems, hyperactivity, and peer problems), school problems (school dissatisfaction, school pressure), and sleep problems (less hours of sleep than recommended, low sleep quality). Problematic SMU was measured by the sum-score of the nine endorsed problematic SMU criteria (min. 0, max. 9). Due to the dichotomous nature of the outcome variables, analyses were conducted using logistic regression. In these regression analyses, we controlled for gender, educational level, age, and ethnic background. To facilitate interpretability, estimates were transformed into *odds ratios* (ORs) that denote the extent to which the odds of, for example, mental health problems increase with the number of endorsed problematic SMU criteria. Good criterion validity of the test score interpretations was established when a higher number of endorsed criteria was associated with higher probabilities of mental, school, and sleep problems.

#### Predictors of Problematic SMU

**Figure 1.**
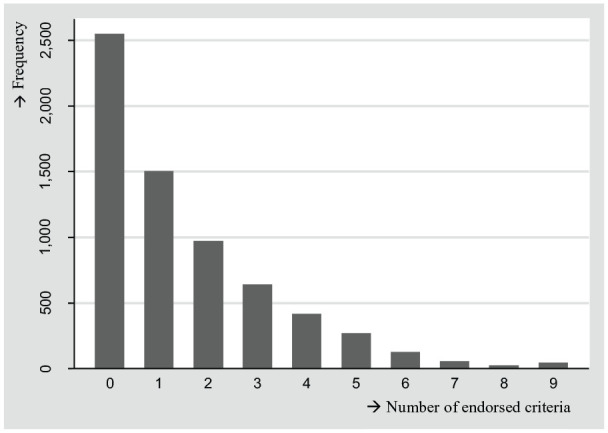
Distribution of the number of endorsed problematic SMU criteria, *N* = 6,609. *Note*. The number of endorsed problematic SMU criteria was measured with the nine-item Social Media Disorder Scale. SMU = social media use.

Following the validation steps, we examined which demographic characteristics (gender, educational level, age, and ethnic background) predicted a higher number of endorsed problematic SMU criteria. Given that this problematic SMU outcome was considered as a count variable with a high number of zero counts (Figure 1), we conducted the analysis using a zero-inflated negative binomial model. We selected this model because it showed better model fit than a zero-inflated Poisson model (chi-bar-square [1] = 428.71, *p* < .001). Furthermore, the zero-inflated negative binomial model showed better fit than an ordinary negative binomial model (*z* = 3.24, *p* ≤ .001). The model was interpreted using *incidence rate ratios* (IRRs), which denote, for example, how much higher the number of endorsed problematic SMU criteria is expected to be for girls relative to boys. IRRs were calculated using boys (gender), highly educated adolescents (educational level), 12-year-olds (age), and native adolescents (ethnic background) as the reference categories.

M*plus* 8.3 (Muthén & Muthén, 2017a) was used to conduct the EFA, CFA, and measurement invariance analysis, using Weighted Least Square Means and Variance Adjusted estimation with a probit regression link and theta parameterization. This estimation method was selected because it provided all fit indices for categorical data that were required for model evaluations. The LCA was also conducted using M*plus* 8.3, but with maximum likelihood estimation with robust standard errors and a logit regression link, as is common for LCA. Stata 14.2 (StataCorp, 2015) was used to conduct Velicer’s MAP analysis using the *minap* package (Soldz, 2002). Analyses related to IRT, criterion validity, and associations between demographic characteristics and problematic SMU were also performed with Stata with the default maximum likelihood estimation. All analyses were conducted with the sample weight and with a cluster correction on school class level to correct for the nested structure of the data. All syntax files are publicly available and may be consulted via https://osf.io/pngw5/.

## Results

### Structural Validity

Table 1 shows that the EFA on the calibration sample identified one factor with an eigenvalue higher than one (4.57), suggesting a one-factor solution. The parallel analysis showed that only the empirical eigenvalue of the first factor exceeded its 95th random data eigenvalue, which also supports a one-factor solution.

**Table 1.** EFA Eigenvalues, Parallel Analysis, and Velicer's MAP Test (calibration sample, *n* = 3,310).

Number of factors	Empirical eigenvalues	Parallel test: 95th percentile of random eigenvalues	Velicer’s MAP test: MAP correlation
0	—	—	0.196
1	4.572	1.103	0.027
2	0.819	1.070	0.048
3	0.746	1.048	0.071
4	0.630	1.028	0.127
5	0.599	1.010	0.222
6	0.562	0.995	0.314
7	0.456	0.978	0.461
8	0.349	0.960	1.000

*Note*. EFA = exploratory factor analysis; MAP = minimum average partial.

Although the model fits of the one-factor (CFI = 0.984; TLI = 0.979; RMSEA = 0.029; SRMR = 0.049), two-factor (CFI = 0.994; TLI = 0.989; RMSEA = 0.021; SRMR = 0.034), and three-factor (CFI = 1.000; TLI = 1.000; RMSEA ≤ 0.001; SRMR = 0.0.016) solutions were all good, the one-factor solution showed the highest quality (Table 2). This is because in the one-factor solution, factor loadings of all items were higher than 0.5, while in the two- and three-factor solutions, there were multiple items with cross-loadings and factor loadings below 0.5. After removal of these items, the factors in the two- and three-factor solutions did not meet the requirement of having at least three items with loadings of 0.5 or higher per factor. Furthermore, the correlations between the factors in the two- and three-factor solutions were high (*r* ≥ 0.59), which suggests that the additional factors strongly overlap and should not be considered as separate factors. The EFA obtained one-factor solution was also found by Velicer’s MAP test, because the one-factor solution showed the lowest average partial correlation (Table 1). The one-factor solution was further evaluated with a CFA using the validation sample. Model fit was good (CFI = 0.983, TLI = 0.977, RMSEA = 0.028, and SRMR = 0.040). Also, the quality of the factor was good, because all nine-factor loadings exceeded 0.5 (Table 2). The one-factor solution was thus confirmed by the CFA using another, randomly selected sample. These results imply that all nine items contributed to one single dimension.

**Table 2.** Results EFA and CFA.

Criterion	During the past year, have you . . .	Calibration sample (*n* = 3,310)							Validation sample (*n* = 3,316)	
		Observed proportion	EFA, rotated factor solutions (β)						Observed proportion	CFA (β)
			One-factor solution	Two-factor solution		Three-factor solution				One-factor solution
			Factor 1	Factor 1	Factor 2	Factor 1	Factor 2	Factor 3		
Preoccupation	. . . regularly found that you can't think of anything else but the moment that you will be able to use social media again?	0.237	0.543*	0.358*	0.232*	0.422*	0.156	0.008	0.282	0.518*
Tolerance	. . . regularly felt dissatisfied because you wanted to spend more time on social media?	0.083	0.729*	0.604*	0.204	0.835*	−0.073	0.020	0.084	0.729*
Withdrawal	. . . often felt bad when you could not use social media?	0.173	0.697*	0.833*	−0.003	0.928*	0.008	−0.183	0.178	0.711*
Persistence	. . . tried to spend less time on social media, but failed?	0.274	0.559*	0.079	0.501*	−0.069	0.699*	0.001	0.235	0.607*
Displacement	. . . regularly had no interest in hobbies or other activities because you would rather use social media?	0.141	0.627*	0.054	0.594*	0.021	0.528*	0.159	0.143	0.660*
Problem	. . . regularly had arguments with others because of your social media use?	0.157	0.748*	−0.068	0.835*	−0.012	0.322*	0.561*	0.160	0.688*
Deception	. . . regularly lied to your parents or friends about the amount of time you spend on social media?	0.129	0.716*	0.006	0.729*	0.011	0.449*	0.358*	0.121	0.684*
Escape	. . . often used social media so you didn't have to think about unpleasant things?	0.297	0.614*	0.243*	0.409*	0.238	0.437*	−0.005	0.284	0.553*
Conflict	. . . had serious conflict with your parents, brother(s) or sister(s) because of your social media use?	0.055	0.795*	−0.002	0.817*	0.103	0.002	0.844*	0.050	0.764*

*Note*. Grey cells depict significant factor loadings at *p* < .05. EFA = exploratory factor analysis; CFA = confirmatory factor analysis.

### Reliability and Item Performance

**Figure 2.**
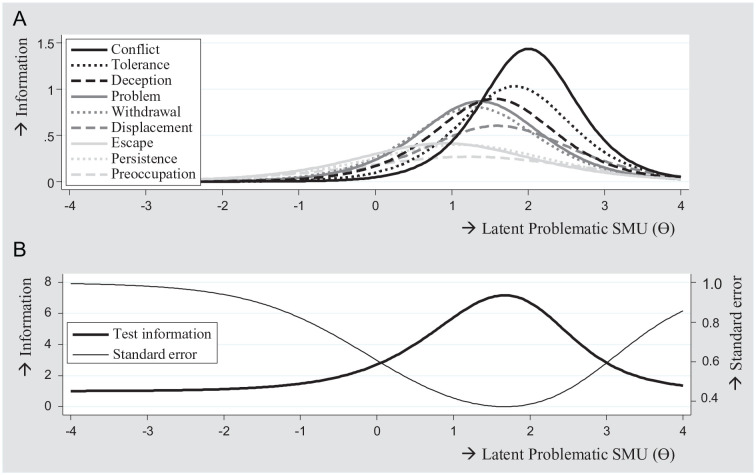
Item information curves (A) and total information curve (B), *N* = 6,626. *Note*. Items in legend were sorted on their discrimination parameter. SMU = social media use.

The ordinal alpha of the one-factor solution was 0.87, which indicates that the internal consistency of the test scores was good. Reliability was further evaluated based on IRT item performance using the two-parameter logistic model. The two-parameter logistic model, which allowed the discrimination parameters to vary, was selected because its fit was better than the one-parameter logistic model, which constrained the discrimination parameters to be equal, χ^2^(8) = 243.67, *p <* .001. IRT models showed that the difficulty parameters of all nine items ranged between 0.91 and 2.01, indicating high difficulty (Baker, 2001). This suggests that the criteria were most likely to be present among adolescents with higher levels of problematic SMU. Discrimination parameters were moderate (1.04 to 1.29; preoccupation, persistence, escape), high (1.55; displacement), or very high (1.80 to 2.40; withdrawal, problem, deception, tolerance, conflict; Baker, 2001). This implies that the criteria had moderate to very high discriminative power to distinguish adolescents with high from those with low levels of problematic SMU. Figure 2A shows that for values at the mean of the latent trait (θ = 0, corresponding to endorsement of ±1 criterion), item “escape” provided the most information. For values that were 1 standard deviation above the mean of the latent trait (θ = 1.00, corresponding to endorsement of ±4 criteria), item “problem” provided the most information. For values 2 standard deviations above the mean (θ = 2.00, corresponding to endorsement of ±7 criteria), item “conflict” provided the most information. Figure 2B shows the information function of the total scale. As can be seen, the scale provided most information on higher values of the latent trait, that is, higher than the mean (θ = 0.00). These findings indicate that test scores were most reliable at moderate to high levels of the scale’s continuum. Total information was highest at θ = 1.68 (corresponding to endorsement of ±6 criteria), which indicates that test scores were most reliable at this value.

### Measurement Invariance

The configural multigroup CFAs all showed good model fit (gender: CFI = 0.983, TLI = 0.977, RMSEA = 0.027, SRMR = 0.039; educational level: CFI = 0.984, TLI = 0.978, RMSEA = 0.026, SRMR = 0.047; age category: CFI = 0.982, TLI = 0.975, RMSEA = 0.028, SRMR = 0.049; ethnic background: CFI = 0.983, TLI = 0.977, RMSEA = 0.027, SRMR = 0.042). All group comparisons showed scalar invariance (gender: ΔCFI = −0.001, ΔRMSEA = −0.001, ΔSRMR = 0.001; educational level: ΔCFI = −0.004, ΔRMSEA = 0.001, ΔSRMR = 0.004; age category: ΔCFI = 0.001, ΔRMSEA = −0.004, ΔSRMR = 0.003; ethnic background: ΔCFI = 0.000, ΔRMSEA = −0.002, ΔSRMR = 0.002), because imposing equality constraints did not substantially deteriorate model fits (Chen, 2007). Thus, the factor loadings and thresholds of all nine items were equal across all group comparisons, which implies measurement invariance across all investigated subpopulations.

### Subgroups of Users

Table 3 shows the results of the LCA. We examined five class solutions, because the five-class solution did not improve model fit relative to the four-class solution (LMR-LRT *p* = .122), which makes estimating additional class solutions redundant (Nylund et al., 2007). The AIC and BIC decreased with each number of increasing classes, indicating that model fit improved with the number of classes (Nylund et al., 2007). However, the classification accuracy of the four- and five-class solutions was lower than 0.7, which is often considered as unacceptable (e.g., Meeus et al., 2010; Reinecke, 2006). This means that there was substantial overlap in adolescents’ item scores between the classes in the four- and five-class solutions, which diminishes the interpretability of the classes (Celeux & Soromenho, 1996). Hence, the two- and three-class solutions were considered more eligible. We selected the three-class solution, which showed a substantial improvement of model fit compared with the two-class solution (ΔAIC = −492.53 and ΔBIC = −424.54).

**Table 3.** Fit Indices and Class Proportions for Five Latent Class Solutions, *N* = 6,626.

C.	Par.	AIC	BIC	LMR-LRT*p*	Entropy	Class size				
						Class 1	Class 2	Class 3	Class 4	Class 5
1	9	51973.96	52035.14			100%				
2	19	47073.14	47202.32	<.001	0.739	73.91%	26.09%			
3	29	46580.61	46777.78	.014	0.726	61.65%	34.75%	3.60%		
4	39	46448.72	46713.87	<.001	0.660	57.39%	29.81%	11.79%	1.01%	
5	49	46378.87	46712.00	.122	0.674	57.39%	29.84%	3.53%	8.18%	1.06%

*Note*. C. = class solution; Par. = number of free parameters; AIC = Akaike information criterion; BIC = Bayesian information criterion; LMR-LRT = Lo-Mendell-Ruben adjusted likelihood ratio test.

A sensitivity analysis was conducted to investigate whether this model selection was robust to conditional dependence of the items. In the one-class solution, 32 out of all 36 possible item correlations were found to be conditionally dependent and specified as such. In the two-class solution, 15 item correlations were specified, and in both the three- and four-class solutions, three item correlations were specified. The LMR-LRT *p* value of the four-class solution was not significant (*p* = .74), and hence no additional classes were estimated. Furthermore, this nonsignificant finding indicated that the four-class solution did not improve model fit relative to the three-class solution. The three-class solution showed the highest Entropy (0.67), and better model fit in terms of the AIC, BIC, and LMR-LRT *p* value than the one- and two-class solutions. Hence, the LCA with conditional dependence also favored the three-class solution. Furthermore, the correlation between adolescents’ class membership based on the three-class solution with conditional dependence and their class membership based on the three-class solution with conditional independence was 0.95, which suggest that the class assignments with and without the imposed assumption were almost identical. These results imply that the model selection is not biased by conditional dependence of the items.

**Figure 3.**
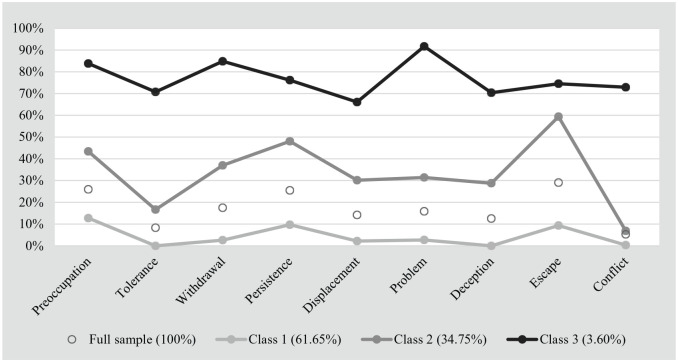
Proportion of positive scores on the nine criteria, by latent class, *N* = 6,626.

Figure 3 illustrates the proportions of positive scores on the nine criteria per class. In Class 1 (61.65% of the sample), for all nine criteria, the proportions of positive scores were lower than in the full sample. In Class 2 (34.75% of the sample), the proportions of positive scores were higher than in the full sample and Class 1 and ranged between 6.88% (“conflict”) and 59.38% (“escape”). In Class 3 (3.60%), the proportions of positive scores were higher than in Class 2 and varied between 66.11% (“displacement”) and 91.70% (“problem”). Given that the proportions of positive scores on the nine criteria were highest in Class 3, followed by Class 2 and Class 1, respectively, we labeled the three classes as *problematic SMU* (Class 3), *risky SMU* (Class 2), and *normative SMU* (Class 1).

**Figure 4.**
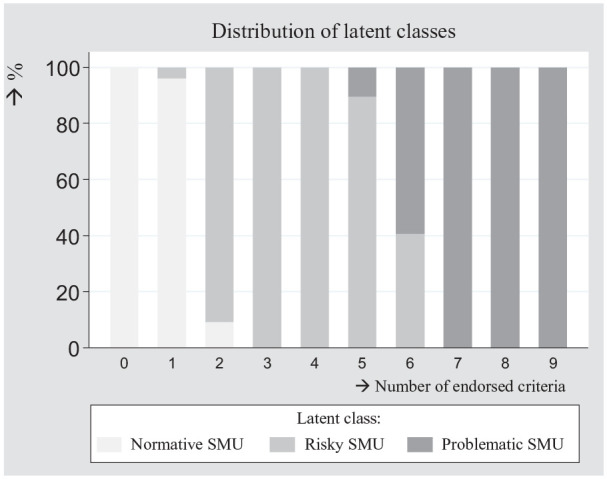
Distribution of latent classes, by the number of endorsed criteria, *N* = 6,626. *Note*. SMU = social media use.

We found that differences in the proportions of endorsed criteria within classes often paralleled the full sample’s differences (e.g., “tolerance” was one of the least endorsed criteria in the full sample and in the three class samples). In other words, we did not observe clear item patterns that distinguished between the three latent classes. Rather, the classes seemed to be distinguished by either high, medium, or low probability of endorsing any of the nine criteria. Therefore, we compared the three classes on adolescents’ number of endorsed criteria. Subsequently, we plotted these scores with the latent classes (Figure 4). In the problematic SMU class, most adolescents (87.07%) endorsed at least six criteria. In the risky problematic SMU class, almost all adolescents (95.11%) endorsed two to five criteria. In the normative problematic SMU class, almost all adolescents (97.86%) endorsed not more than one criterion. These results suggest that subgroups may be distinguished by the number of endorsed criteria rather than by the presence of a particular set of criteria or criterion.

### Criterion Validity

Table 4 reports the associations between problematic SMU and mental, school, and sleep problems. The higher the number of endorsed criteria, the higher the probability of reporting problems related to mental health, school, and sleep, *OR*_range_ = 1.18 (low sleep duration) to 1.40 (conduct problems), *p* < .001. In separate models, we additionally examined the extent to which subgroups of users reported differences in mental, school, and sleep problems. Based on the findings from the LCA (Figure 4), we distinguished normative users (endorsement of not more than one criterion), risky users (endorsement of two to five criteria), and problematic users (endorsement of six to nine criteria). Subgroup differences were investigated while controlling for demographic characteristics and with a Bonferroni correction. Results in Table 4 show that risky users were more likely to report mental health, school, and sleep problems than normative users, *OR*_range_ = 1.63 (peer problems) to 2.81 (emotional problems), *p* < .001. To an even greater extent, problematic users were more likely to report problems related to mental health, school, and sleep, than normative users, *OR*_range_ = 2.47 (low sleep duration) to 8.44 (conduct problems), *p* < .001. Furthermore, post hoc pairwise comparisons showed that problematic users had a higher probability of reporting mental health problems, school problems, and low sleep quality than risky users. Problematic and risky users were equally likely to report low sleep duration.

**Table 4.** Logistic Regression Results, Problems Related to Mental Health, School, and Sleep, *N* = 6,626.

	Mental health problems															
	Emotional problems^a^				Conduct problems^b^				Hyperactivity^c^				Peer problems^d^			
	Β	*SE*	*OR*	M%	Β	*SE*	*OR*	M%	Β	*SE*	*OR*	M%	Β	*SE*	*OR*	M%
Number of endorsed criteria	0.31***	0.02	1.36	3.99	0.34***	0.02	1.40	3.38	0.21***	0.02	1.24	2.92	0.20***	0.02	1.22	2.10
Normative SMU (max. one criterion)	ref. (a)			10.57	ref. (a)			7.65	ref. (a)			12.77	ref. (a)			10.04
Risky SMU (two to five criteria)	1.03*** (b)	0.07	2.81	24.93	1.00*** (b)	0.08	2.73	18.44	0.68*** (b)	0.06	1.98	22.51	0.49*** (b)	0.08	1.63	15.39
Problematic SMU (six to nine criteria)	1.74*** (c)	0.15	5.70	40.23	2.13*** (c)	0.16	8.44	41.13	1.34*** (c)	0.15	3.83	35.91	1.27*** (c)	0.16	3.56	28.43
	School problems								Sleep problems							
	School dissatisfaction^e^				Perceived school pressure^f^				Lower sleep duration than recommended^g^				Low sleep quality^h^			
	Β	*SE*	*OR*	M%	Β	*SE*	*OR*	M%	Β	*SE*	*OR*	M%	Β	*SE*	*OR*	M%
Number of endorsed criteria	0.17***	0.02	1.19	3.10	0.24***	0.02	1.27	5.45	0.16***	0.02	1.18	3.35	0.25***	0.02	1.28	3.93
Normative SMU (max. one criterion)	ref. (a)			19.61	ref. (a)			29.57	ref. (a)			24.39	ref. (a)			15.09
Risky SMU (two to five criteria)	0.50*** (b)	0.06	1.64	28.61	0.75*** (b)	0.06	2.11	46.94	0.55*** (b)	0.06	1.73	35.76	0.78*** (b)	0.07	2.18	27.96
Problematic SMU (six to nine criteria)	0.95*** (c)	0.15	2.58	38.61	1.25*** (c)	0.14	3.48	59.38	0.90*** (b)	0.15	2.47	44.36	1.58*** (c)	0.16	4.85	46.30

*Note*. Rows with different letters denote significant group differences at *p* < .05 with Bonferroni correction. SMU = social media use; *OR* = odds ratios from multivariate logistic regression, controlled for gender, age, education level, and ethnic background; *SE* = standard error; M% = margin, that is, expected probability while holding all covariates at their means; Ref. = reference category.

a Borderline/abnormal range of emotional problems (Score 5 or higher out of 10). ^b^Borderline/abnormal range of conduct problems (Score 4 or higher out of 10). ^c^Borderline/abnormal range of hyperactivity (Score 7 or higher out of 10). ^d^Borderline/abnormal range of peer problems (Score 4 or higher out of 10). ^e^Does not like school very much or not at all. ^f^Feels some or a lot pressure by schoolwork. ^g^Average sleep duration on weekdays does not meet the age-specific recommendation. ^h^An average score of 3.5 or higher on five items from the Groningen Sleep Quality Scale.

* *p* < .05. **p < .01. ***p < .001.

To facilitate interpretability, we transformed *OR*s into *marginal effects* (M), which denote effect sizes in terms of probabilities (Williams, 2012). Table 4 shows that for each increase in the number of endorsed criteria, the probability of reporting mental, school, and sleep problems increases with 2.10 (peer problems) to 5.45% (perceived school pressure). The subgroups differed most in emotional and conduct problems: Compared with normative users (10.57% and 7.65%, respectively), risky users were more than twice as likely to report emotional problems and conduct problems (24.93% and 18.44%, respectively), and problematic users were four to five times more likely to report emotional and conduct problems (40.23% and 41.13%, respectively).

In sum, these findings confirm criterion validity of the test score interpretations, because the higher the level of problematic SMU, the higher the probability of problems related to mental health, school, and sleep. Also, as compared to adolescents in the normative SMU-subgroup, adolescents in the problematic SMU subgroup reported more mental health, school, and sleep problems, followed by adolescents in the risky SMU-subgroup.

### Predictors of Problematic SMU

Table 5 shows the associations between adolescents’ demographic characteristics and their number of endorsed problematic SMU criteria (*p* values were adjusted with Bonferroni corrections). For girls, the number of endorsed criteria was 1.28 times higher than for boys. For lower and medium educated adolescents, the number of endorsed criteria was 1.42 and 1.27 times higher than for higher educated adolescents. Post hoc pairwise comparisons showed that lower educated adolescents also endorsed more criteria than medium educated adolescents. Compared with 12-year-olds, the number of endorsed criteria was 1.16 times higher for 15-year-olds. Post hoc pairwise comparisons showed that 12- and 15-year-olds were the only age groups that differed significantly in the number of present criteria. For adolescents with a non-Western immigrant background, the number of endorsed criteria was 1.20 higher than for native adolescents. Post hoc pairwise comparisons showed no other differences by ethnic background.

**Table 5.** Zero-Inflated Negative Binomial and Multinomial Regression, Demographic Characteristics and Problematic SMU, *N* = 6,626.

	Zero-inflated negative binomial			Multinomial (ref. = normative SMU, max. one criterion)							
	Number of endorsed criteria			Risky SMU (two to five criteria)				Problematic SMU (six to nine criteria)			
	Β	*SE*	IRR	Β	*SE*	*OR*	M%	Β	*SE*	*OR*	M%
*Gender*											
Boys	ref.			ref.			28.86	ref.			2.89
Girls	0.25***	0.03	1.28	0.58***	0.06	1.79	41.29	0.56***	0.14	1.75	4.06
*Educational level*											
High (pre-university)	ref. (a)			ref. (a)			30.57	ref. (a)			1.81
Medium (general higher)	0.24*** (b)	0.04	1.27	0.30** (b)	0.08	1.36	36.61	0.82** (b)	0.23	2.27	3.63
Low (pre-vocational)	0.35*** (c)	0.04	1.42	0.31** (b)	0.08	1.36	36.18	1.14*** (b)	0.22	3.14	4.95
*Age*											
12	ref. (a)			ref. (a)			30.81	ref. (a)			2.55
13	0.14 (ab)	0.05	1.15	0.29* (b)	0.09	1.34	36.98	0.36 (a)	0.22	1.43	3.27
14	0.14 (ab)	0.05	1.16	0.25 (ab)	0.09	1.29	35.74	0.61 (a)	0.23	1.84	4.23
15	0.15* (b)	0.05	1.16	0.29* (b)	0.09	1.34	36.67	0.55 (a)	0.22	1.73	3.93
16	0.05 (ab)	0.05	1.05	0.12 (ab)	0.10	1.12	33.04	0.33 (a)	0.23	1.40	3.40
*Ethnic background*											
Native	ref. (a)			ref. (a)			34.19	ref. (a)			3.10
Non-Western	0.19* (b)	0.04	1.20	0.14 (a)	0.08	1.15	36.63	0.56** (b)	0.17	1.75	5.05
Other Western	0.14 (ab)	0.06	1.15	0.16 (a)	0.12	1.17	36.92	0.60 (ab)	0.29	1.82	5.20

*Note*. Rows with different letters denote significant group differences at *p* < .05 with Bonferroni correction. SMU = social media use; *SE* = standard error; IRR = incidence rate ratio; *OR* = odds ratio; M% = margin, that is, expected probability while holding all covariates at their means; Ref. = reference category.

* *p* < .05. **p < .01. ***p < .001.

In addition, we repeated previous analyses, but used risky and problematic SMU as outcome conducting multinomial regression (using normative SMU as the reference category). Table 5 shows that girls and adolescents who attended low or medium education were more likely to report risky SMU and problematic SMU than boys and adolescent who attended high education, respectively. For example, 4.06% of all girls were likely to report problematic SMU, compared with 2.89% of all boys. Compared with 12-year-olds, 13- and 15-year-olds had a higher probability of reporting risky SMU (30.81% versus 36.98% and 36.67%, respectively). Problematic SMU did not vary significantly by age. Risky SMU did not vary across ethnic background, but non-Western adolescents had a higher probability of reporting problematic SMU compared with native adolescents (5.05% vs. 3.10%).

## Discussion

Using a large-scale, nationally representative sample of Dutch adolescents, the present study demonstrated good psychometric properties for the SMD scale (Van den Eijnden et al., 2016), which measures problematic SMU. Multiple assessments of structural validity showed a solid unidimensional factor structure, whereby all nine items substantially contributed to the factor. The test scores showed good internal consistency, but they were most reliable at higher levels of the scale’s continuum. The factor structure was measurement invariant across gender, educational level, age, and ethnic backgrounds. The data yielded three subgroups of users that were distinguished by low, medium, and high proportions of positive scores on all criteria rather than on particular sets of criteria. These subgroups were labelled as normative, risky, and problematic users, respectively. Furthermore, the criterion validity of the test score interpretations was good: In line with previous research, a higher level of problematic SMU was associated with a higher probability of reporting mental health problems, school problems, and sleep problems. Furthermore, problematic users reported the most mental health, school, and sleep problems, followed by risky and normative users. Girls, low- and medium-educated adolescents, 15-year-olds, and non-Western adolescents endorsed more problematic SMU criteria than boys, high-educated adolescents, 12-year-olds, and native adolescents, respectively.

The finding that the dimensionality assessments identified one underlying factor and that all nine items substantially contributed to the factor implies that the scale measured one construct as intended, and that computing a sum-score from all nine items to assess problematic SMU is valid. It has been argued that some items may identify problematic (social media) behaviors more strongly than others (Kardefelt-Winther et al., 2017). Although the factor loadings of the nine items varied, the small observed differences in their strengths do not support this theory-driven argument. In addition, although the SMD scale was developed as a unidimensional scale, arguably, a multidimensional factor structure would have been plausible. For example, one may argue that some criteria relate to a behavioral dimension of problematic SMU (e.g., conflict, problem), whereas others to a cognitive (e.g., preoccupation, tolerance). The finding that the unidimensional factor structure was most adequate implies that despite the potential conceptual overlap between particular criteria, together the nine criteria reflect one underlying dimension. However, to consolidate this suggestion, additional exploratory dimensionality tests on data from an extended version of the SMD scale, which uses more items per criterion (Lemmens et al., 2015; Van den Eijnden et al., 2016), are warranted.

The finding that the factor structure was measurement invariant suggests that the test scores can be used to reliably compare problematic SMU sum-scores across gender, educational levels, age categories, and ethnic backgrounds. This is an important finding since to our knowledge, no previous studies have investigated measurement invariance of any problematic SMU-scale across these four subpopulations using nationally representative data on adolescents. As a result, it remained unclear whether prevalence differences reported in previous research (Bányai et al., 2017; Ho et al., 2017; Mérelle et al., 2017) were biased by varying measurement properties across subpopulations.

The criterion validity analysis showed that the higher the number of endorsed problematic SMU criteria, the higher the probability of reporting problems related to mental health, school functioning, and sleep, confirming good criterion validity of the test score interpretations. Problematic users typically experience unpleasant feelings such as stress or anxiety when SMU is restricted, which may induce mental health problems. Also, the loss of control over SMU may make it difficult to regulate schoolwork responsibilities, which may increase school problems. In addition, being preoccupied with social media or feeling a constant urge to go online may be associated with sleep difficulties. Or conversely, adolescents with problems related to their mental health, school functioning, or sleep may engage in problematic SMU to cope with their problems (Kuss & Griffiths, 2017). Longitudinal research is warranted to examine the directionality of these associations.

In addition, in the criterion validity analysis we also examined the extent to which mental health, school, and sleep problems differed between three subgroups: normative users (endorsement of max. one criterion), risky users (endorsement of two to five criteria), and problematic users (endorsement of six to nine criteria). Although these thresholds for classification were based on observed patterns in the data, research using clinical samples is required to examine whether this classification is justified. Nevertheless, the criterion validity analysis supports the validity of the classification, because the three subgroups differed significantly on mental health, school, and sleep problems, with problematic users being most at risk, followed by risky users and normative users. Furthermore, the finding that risky users were more likely to report problems related to mental health, school, and sleep emphasizes that it is important to study moderate levels of problematic SMU and not only the highest levels, as the presence of a few criteria already seems indicative of problems in several important life domains.

In line with former research (Bányai et al., 2017; Ho et al., 2017; Mérelle et al., 2017), our study showed that the number of endorsed problematic SMU criteria was highest among girls, low-educated adolescents, and non-Western adolescents. In addition, the number of endorsed criteria peaked at 15 years, suggesting that the association between age and problematic SMU was nonlinear. This nonlinear association may explain why previous research on problematic SMU in adolescents found only a small effect size of age or no age differences at all (Bányai et al., 2017; Ho et al., 2017; Mérelle et al., 2017).

There may be several reasons why girls, lower educated, 15-year-olds, and non-Western adolescents reported higher levels of problematic SMU. Girls may find it more important to maintain and expand social relationships and to express or validate their thoughts and feelings than boys (Kuss & Griffiths, 2011, 2017). This may make girls more vulnerable to developing problematic SMU, as social media facilitates fulfilling these needs (Kuss & Griffiths, 2011, 2017). In addition, Dutch adolescents with a low educational level or with a non-Western background are relatively likely to come from low socioeconomic status families (CBS, 2017, 2018). Adolescents with low socioeconomic status backgrounds are more sensitive to engaging in risky behavior in general than adolescents with high socioeconomic status backgrounds, possibly related to lower support from family, cognitive challenges, or limited self-control (Inchley et al., 2016; Stevens et al., 2018). Similarly, adolescents with a low educational level or with a non-Western background may be more sensitive to developing problematic SMU. Furthermore, the finding that the level of problematic SMU was highest among 15-year-olds implies that there may be an increased risk of problematic SMU during this stage of adolescence. The popularity of social media during adolescence may reach its peak at this age, which may make social media harder to resist. However, empirical research is required to examine the mechanisms underlying the differences found in the present study.

In addition, the observed proportions of positive scores on the problematic SMU criteria were rather low (<30%). Consequently, the scale’s sum-scores showed a skewed distribution, indicating that many adolescents did not endorse any criteria, and a minority endorsed many criteria. This finding suggests that higher levels of problematic SMU are relatively uncommon, which is in line with previously reported prevalence rates of problematic SMU and other problematic internet-related behaviors, including internet gaming disorder and internet addiction (Andreassen, 2015; Kuss et al., 2014; Lemmens et al., 2015). While intense SMU, indicated by very frequent use of social media, is common among contemporary adolescents (Anderson & Jiang, 2018), scholars emphasize that a rather small proportion of social media users may adopt addiction-like behavior regarding their SMU, such as loss of control or interference with daily activities (Griffiths, 2013; Kardefelt-Winther et al., 2017). Hence, the distribution of the sum-scores as observed in the present study supports the validity of the test score interpretations.

### Strengths, Limitations, and Future Directions

This study has important strengths related to the nationally representative character of the data and the number and variety of psychometric tests supporting the reliability and validity of the SMD scale scores and interpretations. Yet there are limitations that constitute promising directions for future research. First, the present study used a large sample of Dutch adolescents aged 12 to 16 years. To establish the generalizability of our findings in other countries and age groups, research using cross-national assessments of the scale among different age categories is required. For instance, a CFA conducted among a sample of 903 Chinese university students aged 18 to 23 years suggested that the scale measured two factors, with the items problem, deception, and conflict representing a separate factor (Fung, 2019), suggesting that the factor structure may differ across age-groups and/or cultures. Second, the nature of the sample did not allow for clinical validation. Research using clinical samples is required to verify whether the SMD scale is feasible as a diagnostic tool that accurately identifies problematic users. Third, IRT-analyses showed that the test scores were most reliable for values above the mean of the latent trait, suggesting that the scale provides more precise estimates at higher levels of problematic SMU than at (more common) lower levels of problematic SMU. Hence, the SMD scale may be most suited to identify moderate to high levels of problematic SMU. This finding is not uncommon for scales that measure exceptional or rare behaviors. For example, validation studies of substance-related disorders and internet gaming disorder scales showed that these scales provide most information at the higher end of the scale’s continuum, that is, for scores that exceed the sample mean (Gomez et al., 2019; Martin et al., 2006; Saha et al., 2006). Fourth, adolescents’ test scores were based on self-reports, which may deviate from their actual behaviors. For example, adolescents may underestimate or overestimate the extent to which their SMU impairs important life domains. Comparing parent and adolescent scores on the SMD scale may provide novel insights into the social reliability of adolescents’ self-reports. Fifth, because the data provided one scale that measured problematic SMU, comparison of the psychometric performance of alternative scales was not possible. The SMD scale distinguishes itself from other scales, such as the BSMAS (Andreassen et al., 2016), by adding the criteria displacement, problems, and deception on top of the six core criteria of addiction. Statistical comparisons of different scales allow researchers to evaluate whether the three additional criteria substantially improve the conceptualization of problematic SMU. Sixth, the criterion validity assessment was limited to measurements related to adolescents’ well-being. Future studies examining the association between adolescents’ intensity of SMU activities and scores on the SMD scale would extend current knowledge on the validity of the scale. In doing so, the use of objective measures of SMU activities collected through, for example, logged social media data (Marengo et al., 2020; Marino et al., 2017) is considered promising.

## Conclusion

The present study has demonstrated that the SMD scale has good psychometric properties. Given its solid factor structure, adequate test score reliability, and good validity of the test score interpretations, the scale is suitable for empirical assessments of problematic SMU among adolescents. The scale thereby facilitates future research on adolescent problematic SMU.
